# Prevalence of Low-Frequency, Antiviral Resistance Variants in SARS-CoV-2 Isolates in Ontario, Canada, 2020-2023

**DOI:** 10.1001/jamanetworkopen.2023.24963

**Published:** 2023-07-21

**Authors:** Calvin P. Sjaarda, Lynette Lau, Jared T. Simpson, Ramzi Fattouh, Mia J. Biondi, Finlay Maguire, Aaron Campigotto, Yujia Feng, Kyla Tozer, Henry Wong, Wilson W. L. Sung, Sean Kim, Christian R. Marshall, Prameet M. Sheth, Robert Kozak

**Affiliations:** 1 Division of Microbiology, Kingston Health Sciences Centre, Kingston, Ontario, Canada; 2 Department of Pathology and Molecular Medicine, Queen’s University, Kingston, Ontario, Canada; 3 Hospital for Sick Children, Toronto, Ontario, Canada; 4 Ontario Institute for Cancer Research, Toronto General Hospital, Toronto, Ontario, Canada; 5 Department of Molecular Genetics, Department of Computer Science, University of Toronto, Toronto, Ontario, Canada; 6 Department of Laboratory Medicine and Pathobiology, Temerty Faculty of Medicine, University of Toronto, Toronto, Ontario, Canada; 7 Li Ka Shing Knowledge Institute, St Michael’s Hospital, Unity Health Toronto, Toronto, Ontario, Canada; 8 School of Nursing, York University, Toronto, Ontario, Canada; 9 Shared Hospital Laboratory, Toronto, Ontario, Canada; 10 Laboratory Medicine and Molecular Diagnostics, Sunnybrook Health Sciences Centre, Toronto, Ontario, Canada

## Abstract

**Importance:**

Nirmatrelvir-ritonavir is an oral antiviral medication that improves outcomes in SARS-CoV-2 infections. However, there is concern that antiviral resistance will develop and that these viruses could be selected for after treatment.

**Objective:**

To determine the prevalence of low-frequency SARS-CoV-2 variants in patient samples that could be selected for by nirmatrelvir-ritonavir.

**Design, Setting, and Participants:**

This retrospective cohort study was conducted at 4 laboratories that serve community hospitals, academic tertiary care centers, and COVID-19 assessment centers in Ontario, Canada. Participants included symptomatic or asymptomatic patients who tested positive for SARS-CoV-2 virus and submitted virus samples for diagnostic testing between March 2020 and January 2023.

**Exposure:**

SARS-CoV-2 infection.

**Main Outcomes and Measures:**

Samples with sufficient viral load underwent next-generation genome sequencing to identify low-frequency antiviral resistance variants that could not be identified through conventional sequencing.

**Results:**

This study included 78 866 clinical samples with next-generation whole-genome sequencing data for SARS-CoV-2. Low-frequency variants in the viral *nsp5* gene were identified in 128 isolates (0.16%), and no single variant associated with antiviral resistance was predominate.

**Conclusions and Relevance:**

This cohort study of low-frequency variants resistant to nirmatrelvir-ritonavir found that these variants were very rare in samples from patients with SARS-CoV-2, suggesting that selection of these variants by nirmatrelvir-ritonavir following the initiation of treatment may also be rare. Surveillance efforts that involve sequencing of viral isolates should continue to monitor for novel resistance variants as nirmatrelvir-ritonavir is used more broadly.

## Key Points

**Question:**

What is the prevalence of low-frequency variants in SARS-CoV-2 isolates from patient samples that could confer resistance to nirmatrelvir-ritonavir?

**Findings:**

In this cohort study, 78 866 SARS-CoV-2 isolates from patients underwent next-generation sequencing, and low-frequency variants were detected in 128 samples (0.16%).

**Meaning:**

These findings suggest that SARS-CoV-2 variants that could be selected for by treatment with nirmatrelvir-ritonavir are rare and that surveillance efforts that involve sequencing of viral isolates should continue to monitor for novel resistance variants.

## Teaser

This cohort study analyzes the prevalence of low-frequency SARS-CoV-2 variants in patient samples that could confer resistance to nirmatrelvir-ritonavir, a common antiviral medication.

## Introduction

The COVID-19 pandemic has resulted in millions of deaths worldwide and continues to be an ongoing global public health challenge. Currently, nirmatrelvir-ritonavir is 1 of 2 oral antiviral medications for the treatment of SARS-CoV-2 and has reduced hospitalizations and deaths^1^; yet, despite the substantial impact of antiviral medications on clinical outcomes, the development of antiviral resistance is an ongoing concern. Because SARS-CoV-2 is an RNA virus, the viral polymerase introduces errors in the genome during replication, and the virus exists as a quasi species in the patient. Therefore, it is unclear whether low-frequency variants may exist that could be selected for by drug pressure, as has been observed with other RNA viruses, such as influenza virus,^2^ hepatitis C virus,^3^ and HIV.^4^

Nirmatrelvir-ritonavir inhibits the main viral protease encoded by the *nsp5* gene,^5^ and ritonavir acts as a boosting agent by inhibiting host cytochrome P450. Studies^6^ have shown that viral rebound has occurred in a subset of treated patients, but it remains unclear whether this could lead to the selection of viruses with reduced antiviral susceptibility. Findings from in vitro studies^7^ have identified variants with the potential to confer resistance to nirmatrelvir-ritonavir without affecting viral fitness.

Importantly, although these variants have been found in clinical isolates,^8^ the prevalence of variants within the Global Initiative on Sharing All Influenza Data database of SARS-CoV-2 sequences was less than 1% among sequenced genomes.^9^ However, this analysis was of the viral consensus sequence, and low-frequency allelic variants that could be selected for could not be detected. There has yet to be a comprehensive clinical evaluation of low-frequency variants that could be selected for during nirmatrelvir-ritonavir treatment. Next-generation sequencing allows for the detection of variants that are present at low frequency. This study examined the prevalence of low-frequency variants within SARS-CoV-2 sequences from clinical samples collected before and after the availability of nirmatrelvir-ritonavir in Ontario, Canada.

## Methods

### Study Design, Setting, and Samples

This cohort study followed the Strengthening the Reporting of Observational Studies in Epidemiology (STROBE) reporting guideline, and did not require ethics board approval or informed consent because the study used deidentified viral sequences collected as part of routine surveillance work, in accordance with 45 CFR §46. This study was a descriptive retrospective analysis of SARS-CoV-2 samples that were submitted for routine diagnostic testing and sequencing. Samples were collected between March 1, 2020, and January 12, 2023, and analyzed at 4 laboratories with tertiary academic hospital centers in Ontario, Canada. All sequencing occurred less than 7 days from the time of collection. These laboratories serve multiple community hospitals, other academic tertiary care centers, as well as COVID-19 assessment centers, and have a total catchment of 5 million Ontarians.

### Whole-Genome Sequencing

Samples that were positive for SARS-CoV-2 using reverse-transcription polymerase chain reaction testing with a cycle threshold less than 30 cycles underwent whole-genome sequencing, as has been previously described.^10^ In a single center, the Illumina platform was used, and data consensus sequences and variants were called using FreeBayes.^11^ The other 3 centers used the Oxford Nanopore Technologies platform, with Nanopore v3 Midnight primers using the native barcode, ARTIC SARS-CoV-2 bioinformatic protocol.^12^ In this context, read length filtering of 400 bp to 700 bp and the V3 primer schema were used. For the Nanopore Midnight primers using rapid barcodes, the Medaka version of the ARTIC pipeline was used and validated alongside the ARTIC protocol. A read length filter of 150 bp to 2000 bp and the Midnight primer schema were used for the rapid barcoding protocol.

### Statistical Analysis

Both platforms’ sample analysis was then assessed for quality using ncov-tools software^13^ version 1.9.1. A list of resistance variants was derived from data provided by the US Food and Drug Administration and a review of the literature.^7,8,14^ Variant files for each sample were filtered to retain variants that were high quality (read depth ≥100 and quality score ≥30), had an allele frequency between 0.1 and 0.9, and were included in the variants of interest list. The nanopore analysis pipelines do not call intrahost variation; thus, we queried the read pileups for support of a minor variant at the positions of interest. The primary outcome was alternate alleles at positions with at least 50 times sequencing depth and an allele frequency between 0.25 and 0.85 that were considered candidate variants for low-frequency variants in the *nsp5* gene. The Fisher exact test was used to determine statistical significance, and a 2-sided *P* < .05 was considered statistically significant. Data analysis was conducted using R statistical software version 4.2.2 (R Project for Statistical Computing). The figure was also done using R version 4.2.2 and packages ggplot2 version 3.4.2 and ggsci version 3.0.0.

## Results

To investigate low-frequency variants, 78 866 sequences from clinical isolates were analyzed, including sequences from current and previous circulating lineages that had been collected before and after the approval of nirmatrelvir-ritonavir in Canada (January 17, 2022). Analysis indicated low-frequency variants at positions linked to drug resistance in the *nsp5* gene in 128 (0.16%) of the isolates. Percentages from each laboratory were similar, with each being less than 1%. Interestingly, a higher than expected count was observed in site B (2 of 372 isolates [0.53%] had low-frequency variants; *P* = .04, Fisher exact test), but this count is likely a result of undersampling bias, because this site had a lower number of sequences (Table). Variation was found at only 33 of the residues identified by the US Food and Drug Administration as being associated with antiviral resistance (eTable in Supplement 1). Additionally, we did not observe more variation at residues that are known to interact with nirmatrelvir-ritonavir compared with other residues (Figure).

**Table.** Low-Frequency Variants From Sequenced SARS-CoV-2 Isolates

Laboratory	Sequenced isolates, No.	Sequencing platform	Isolates with low-frequency variants, No. (%)	Collection period
A	22 635	Illumina	44 (0.19)	April 2021 to September 2022
B	372	Oxford Nanopore Technologies	2 (0.53)	March 2020 to March 2021
C	8283	Oxford Nanopore Technologies	7 (0.08)	June 2022 to January 2023
D	47 576	Oxford Nanopore Technologies	75 (0.15)	September 2021 to January 2023
Total	78 866	NA	128 (0.16)	NA

Abbreviation: NA, not applicable.

**Figure.**
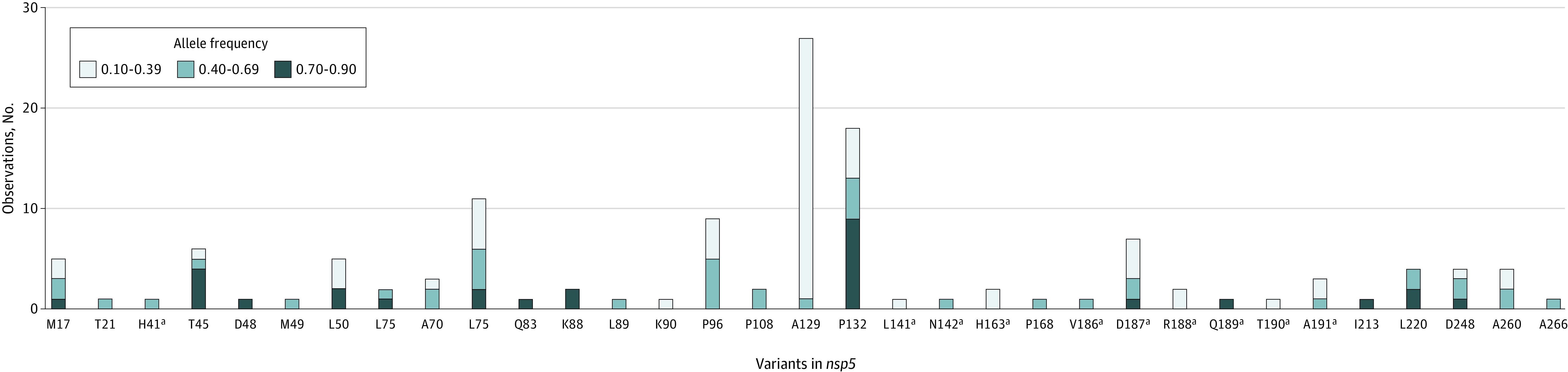
Low-Frequency SARS-CoV-2 Variants Observed in Main Viral Protease Low-frequency variants identified by next-generation sequencing at key residues in the *nsp5* gene that are associated with resistance to nirmatrelvir-ritonavir are shown. Frequency of detection is indicated by color. ^a^Denotes residues that have contact with nirmatrelvir-ritonavir.

Only 1 sample showed variation at residue H41, which is part of the catalytic dyad and is associated with drug binding.^15^ Variation at T190, a residue that interacts with nirmatrelvir-ritonavir, was also noted in 1 sample. A total of 2 samples showed variants at M49 and N142, respectively, which have been associated with a decrease in activity of nirmatrelvir-ritonavir without a significant loss of protease activity.^16^ No variants were seen at residues P252 or T304, both of which have been shown to reduce nirmatrelvir-ritonavir activity in vitro.^17^ A total of 4 samples had low-frequency variants at L50; variants at this position have been shown to confer low-level resistance to nirmatrelvir-ritonavir.^7^ A single isolate did show variation at T21, which is also associated with decreased antiviral susceptibility.^7^

## Discussion

Currently, nirmatrelvir-ritonavir is 1 of 2 oral antiviral treatments for SARS-CoV-2. This cohort study found that naturally occurring low-frequency variants in *nsp5* are rare. Moreover, among the variants detected, no single variant predominated. Studies^18^ suggest that only variants present at more than 15% are likely to represent viral adaptation in the face of selective drug pressure. The paucity of these variants suggests it may be difficult for resistant variants to be selected, and this may be a contributing factor to why resistance to nirmatrelvir-ritonavir has not yet been observed. A high degree of conservation of the viral protease is likely necessary because of its critical role in the viral life cycle.^19,20^ Interestingly, recent studies have estimated that the nonsynonymous variant rate for the protease is more than 10-fold lower than that for the viral polymerase.^9^

Cell culture studies^7^ identified the variants L50F and E166A as conferring resistance to nirmatrelvir-ritonavir, as well as T21I and T304I. Moreover, the presence of these variants in combination increased half maximal effective concentration values, without a complete loss of viral fitness^7^; however, there appears to be a need for compensatory variants.^17^ In our data, low-frequency variants at these positions were rare, and notably, combinations of low-frequency variants at multiple positions (eg, L50 and E166) were not detected. This suggests that there is a potential fitness cost to these variants that would not be observed in cell culture. The viral protease plays a role in moderating the host immune response, and these variants may affect that activity. This hypothesis is supported by our findings that low-frequency variants were found more often in regions outside the binding pocket and at sites that would be less likely to have a major effect on protease activity. Additionally, the use of nirmatrelvir-ritonavir has been relatively limited in Ontario because of an initially limited supply of the drug, government restrictions, and prescribing guidelines,^21^ which could have limited the selective pressure placed on the virus and may partially account for the paucity of antiviral resistance variants observed.

### Limitations

A limitation of this study is that variants detected at low frequencies could be due to artifacts of the sequencing process. Nonetheless, we included these data to highlight that even with this caveat, variations at these key positions are rare. Additionally, there were no clinical histories of the patients from which the samples were derived, and it cannot be discounted that some patients may have received antiviral treatment before testing. Also, patient samples with low viral loads were unable to be sequenced owing to assay limitations and, therefore, could not be included in our analysis. Future studies are needed that include relevant patient medical histories, as well as improved methods for sequencing of isolates from cases with low viral loads.

## Conclusions

In conclusion, our data suggest that low-frequency variants of SARS-CoV-2 at the population level are rare. Surveillance efforts that involve sequencing of viral isolates should continue to monitor for novel resistance variants as nirmatrelvir-ritonavir is used more broadly.

## Notes

Supplement 1. **eTable.** Residues in NSP5 Viral Protease in Contact With Nirmatrelvir

Supplement 2.
Data Sharing Statement
