# Supplementary material for: Prevalence of Low-Frequency, Antiviral Resistance Variants in SARS-CoV-2 Isolates in Ontario, Canada, 2020-2023
Source: JAMA Netw Open. 2023 Jul 21;6(7):e2324963. doi: 10.1001/jamanetworkopen.2023.24963 (PMC10362469; doi:10.1001/jamanetworkopen.2023.24963)
Supplement: Supplement 1. — eTable. Residues in NSP5 Viral Protease in Contact With Nirmatrelvir [file jamanetwopen-e2324963-s001.pdf]

Supplemental Online Content

Sjaarda CP, Lau L, Simpson JT, et al. Prevalence of low-frequency, antiviral resistance variants in SARS-CoV-2 isolates in Ontario, Canada, 2020-2023. *JAMA Netw Open*. 2023;6(7):e2324963. doi:10.1001/jamanetworkopen.2023.24963

eTable. Residues in NSP5 Viral Protease in Contact With Nirmatrelvir

This supplemental material has been provided by the authors to give readers additional information about their work.

© 2023 Sjaarda CP et al. *JAMA Network Open.*

eTable. Residues in NSP5 Viral Protease in Contact With Nirmatrelvir	
Amino Acid	Contact with Nirmatrelvir
M17	
T21	
H41	Yes
T45	
D48	
M49	Yes
L50	
L58	
A70	
L75	
Q83	
K88	
L89	
K90	
P96	
P108	
A129	
P132	
L141	Yes
N142	Yes

© 2023 Sjaarda CP et al. *JAMA Network Open.*

H163	Yes
P168	
V186	Yes
D187	Yes
R188	Yes
Q189	Yes
T190	Yes
A191	Yes
I213	
L220	
D248	
A260	
A266	

© 2023 Sjaarda CP et al. *JAMA Network Open.*
