# Supplementary material for: Prevalence of Low-Frequency, Antiviral Resistance Variants in SARS-CoV-2 Isolates in Ontario, Canada, 2020-2023
Source: JAMA Netw Open. 2023 Jul 21;6(7):e2324963. doi: 10.1001/jamanetworkopen.2023.24963 (PMC10362469; doi:10.1001/jamanetworkopen.2023.24963)
Supplement: Supplement 2. — Data Sharing Statement [file jamanetwopen-e2324963-s002.pdf]

Data Sharing Statement

Sjaarda. Prevalence of Low-Frequency, Antiviral Resistance Variants in SARS-CoV-2 Isolates in Ontario, Canada, 2020-2023. *JAMA Netw Open*. Published July 21, 2023. doi:10.1001/jamanetworkopen.2023.24963

Data
Data available: Yes
Data types: Data (not involving human participants)
How to access data: Data will be made available on GISAID or on appropriate webserver (eg. NCBI) rkozak@shn.ca
When available: With publication

Supporting Documents
Document types: None

Additional Information
Who can access the data: researchers whose proposed use of the data has been approved
Types of analyses: Analysis approved by the research team
Mechanisms of data availability: signed data access agreement
